# Thiacloprid Exposure Induces Oxidative Stress, Endoplasmic Reticulum Stress, and Apoptosis in the Liver of *Mauremys reevesii*

**DOI:** 10.1002/ece3.70936

**Published:** 2025-02-04

**Authors:** Shuqin Lin, Yunjuan Xiao, Siyu Li, Liyan Tang, Haitao Shi, Meiling Hong, Li Ding

**Affiliations:** ^1^ Ministry of Education Key Laboratory for Ecology of Tropical Islands, Key Laboratory of Tropical Animal and Plant Ecology of Hainan Province College of Life Sciences, Hainan Normal University Haikou China

**Keywords:** aquatic turtles, hepatic toxicity, neonicotinoid insecticides, pesticide exposure

## Abstract

Among neonicotinoid insecticides, thiacloprid (THI) is extensively utilized in agricultural practices, which poses a potential toxicity risk to aquatic fauna. Turtles, integral to aquatic ecosystems, have not yet been comprehensively assessed for their vulnerability to THI exposure. In this study, we aimed to evaluate the effects of THI on oxidative stress, endoplasmic reticulum stress (ERS), and apoptosis in aquatic turtles. We categorized *Mauremys reevesii* into three groups: a control group and two experimental groups exposed to environmentally relevant (4.5 μg/mL) and high (15 mg/mL) concentrations of THI, respectively. Transcriptome analysis revealed that genes significantly associated with the elimination of superoxide radicals, organelle inner membrane functions, peroxiredoxin activity, and apoptotic pathways were abundantly expressed in the high‐concentration THI group. Notably, exposure to high concentrations of THI led to a marked increase in glutathione peroxidase (GPX) and superoxide dismutase (SOD) activities, whereas catalase (CAT) activity declined and malondialdehyde (MDA) levels rose, indicating the presence of oxidative stress. Moreover, THI upregulated the expression of the ER stress marker GRP78. Simultaneously, the mRNA levels of pivotal unfolded protein response genes, including AFT6, AFT4, IRE1α, CHOP, XBP1, and eIF2α, were significantly elevated in response to THI exposure. Furthermore, high concentrations of THI significantly activated the activities of caspase‐3, caspase‐8, and caspase‐9 enzymes in the liver tissue. The expression of anti‐apoptotic gene Bcl‐2 was downregulated, whereas the pro‐apoptotic genes Bax and caspase‐3 were upregulated, leading to an increase in hepatic apoptotic cells following THI exposure. Collectively, our study indicates that THI can induce hepatic damage in turtles through the promotion of oxidative stress, ERS, and apoptosis. These findings gain a deeper understanding of the toxic effects of THI on keystone species in aquatic ecosystems, thereby improving our overall understanding of their environmental impacts.

## Graphical abstract

Thiacloprid (THI), widely used for crop protection and pest control, is easily residual and accumulated in the environment because of long half‐life and high leaching effect. The detectable rate of THI in soil and water is high, which can lead to the accumulation in organisms and cause toxic effects through sustained exposure to humans and nontargeted organisms. Turtles have the characteristics of bioaccumulation and biomagnification because of their long‐life history in water environment. The adverse effects of THI on aquatic turtles in this study are of great significance to evaluate the toxic effects of thiacloprid on aquatic organisms and environment.

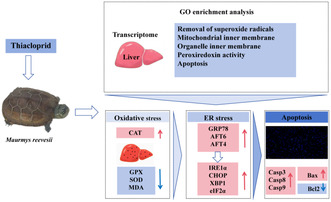

## 1 Introduction

Agricultural pollution is a major source of environmental contamination. This includes soil and water pollution caused by the excessive use of pesticides and fertilizers (Liu 2024). When pesticides are applied to crops, they are washed into the soil by rainwater, contaminating surface waters and potentially contaminating groundwater sources (Bezerra et al. 2020; Chen et al. 2019). The widespread use of pesticides poses potential risks to the ecological environment, human health, and animal welfare. An increasing body of research indicates that pesticide pollution is detrimental to aquatic ecosystems and the health of aquatic organisms. It poses acute toxic risks to nontarget organisms in the environment (Chen et al. 2019) and also affects their reproduction, behavior, physiology, and development (Mengoni Goñalons and Farina 2015; Yin et al. 2014).

Thiacloprid (THI) is a neonicotinoid pesticide with the structural formula [3‐[(6‐chloropyridin‐3‐yl)methyl]‐1,3‐thiazolidin‐2‐ylidene]cyanamide. It can selectively act on nicotinic acetylcholine receptors (nAChRs) in the insect nervous system. THI serves as a crop protection agent for seed treatment and pest control (Jeschke et al. 2011). It is effective against a broad spectrum of target insects and is widely used in vegetables, fruit trees, cotton, rice, tea, and other crops, making it one of the most extensively used insecticides worldwide (Singh et al. 2024). However, THI has strong stability and environmental accumulation, and does not degrade easily even under heavy rain conditions, showing good rain tolerance and photostability (Chen et al. 2021). A growing body of research indicates that residues of THI in the environment are more prevalent than expected and persist for long periods. For example, THI was detected at concentrations of 0.0178 μM in the Elbe River near Hamburg (Süß et al. 2006), 0.0054 μM in rivers near Sydney (Sanchez‐Bayo and Hyne 2014), and 0.00063 μM in the Sousa River in Portugal (Sousa et al. 2019). Residues of THI can severely affect aquatic life including neurotoxicity, immunotoxicity, hepatotoxicity, nephrotoxicity, and reproductive problems, then cause significant ecological damage. For instance, THI exposure causes cognitive impairment, hippocampal structural damage, and liver abnormalities (Singh et al. 2024); THI has been found to cause severe DNA damage in zebrafish ( *Danio rerio* ), activate apoptotic pathways, induce liver damage (Xie et al. 2022; Yavuz Türel, Toğay, and Aşcı Çelik 2023), inhibit embryonic development, and growth rate, and antioxidant capacity in carp ( *Cyprinus carpio* ), and damage the digestive glands and gills (Velisek and Stara 2018).

Aquatic reptiles play a key role in maintaining aquatic and agricultural ecosystems, which are crucial for ecological balance and food security (Cao et al. 2021). They serve as exemplary subjects and key representatives in the study of the origin of tetrapods and the transition from aquatic to terrestrial life, and occupy an important position in the evolutionary history of vertebrates (Li et al. 2017). Within the food chain and ecosystems, reptiles also act as vital trophic intermediates, helping to maintain ecosystem integrity and biodiversity. Consequently, they are considered important indicators of environmental health and serve as important early warning systems for monitoring environmental change (Pounds et al. 2006; Wake 2007; Gonkowski and Ochoa‐Herrera 2024). However, the impact of environmental pollution on reptiles has been largely overlooked in scientific research. Aquatic turtles, which belong to the family of reptiles, are essential components of ecosystems with a long evolutionary history and are often found in habitats such as agricultural waters. Thus, they are ideal candidates for studying the long‐term toxicological effects of agricultural pollution (Huang et al. 2021; Ding et al. 2023). Aquatic turtles are also prone to exposure to pesticides and are threatened by them.

THI is slowly degraded in different environments, has strong environmental accumulation, and may be enriched in organisms (Han et al. 2023). The liver is an important organ to evaluate the enrichment and toxicity effects of this chemical. In addition, the liver is the main antioxidant organ responsible for removing reactive oxygen species and reactive nitrogen species from the body (Li et al. 2015). Pesticide exposure can affect the oxidative state of the liver and lead to oxidative stress, which may cause liver injury (Hernández et al. 2013; Coremen et al. 2022). Previous studies have shown that the duration of exposure in THI‐induced toxicity experiments was 4 weeks in zebrafish (Xie et al. 2022), 35 days in common carp ( *Cyprinus carpio* ) (Velisek and Stara 2018), 6 weeks in Japanese quail (Han et al. 2023). For the turtle growth cycle, the growth and metabolic rates are relatively slow, so a longer period of time is needed to observe and evaluate the effects of pesticides. In this study, we selected the representative turtle species *Mauremys reevesii* as a model and the liver as the target tissue with an exposure time of 5 weeks to investigate the toxic effects of THI exposure on oxidative stress, endoplasmic reticulum stress (ERS), and apoptosis, to elucidate the toxic effects of THI on freshwater turtles.

## 2 Materials and Methods

### 2.1 Animals

The turtles utilized in this experiment were sourced from Haikou Hongwang Agricultural Breeding Co. Ltd. (Haikou, China) and subsequently housed in the animal breeding facility of the College of Life Science at Hainan Normal University. Animals were kept at a temperature of 29°C ± 1°C, water pH 7.45 ± 0.25, and light duration of 12 h light–dark cycle. According to the optimal light intensity for aquatic animal growth, the light intensity was selected to be 50 lx (Huang, Zhao, and Wang 2007; Biswas and Takii 2016; Bonvini et al. 2016). They were acclimated in the breeding room for over 1 month before the commencement of the experiment. Ethical approval for the animal experiment was granted by the Animal Research Ethics Committee of the Hainan Provincial Education Center for Ecology and Environment, Hainan Normal University (HNECEE‐2023‐005).

### 2.2 Drug Exposure Methods

The healthy adult female turtles, 4 years of age with a relatively uniform body size, were selected (average body weight of 234.4 ± 41.4 g). THI was obtained from Jiangsu Limin Chemical Co. Ltd. THI is soluble in water and was directly configured with water. We refer to the methods of Xie et al. (2022) and Shi et al. (2023) acute toxicity tests on fish and bees to establish the median lethal concentration (LC_50_) for exploring the effects of THI on turtles. The turtles were exposed to six different concentrations of THI (15.265, 31.25, 62.5, 125, 250, and 500 mg/L) and a blank control. Each concentration group comprised six turtles, and the 96‐h acute toxicity tests were conducted. GrapPad Prism 9.0 statistical software was used to calculate and plot the median lethal concentration (LC_50_) of THI, and the LC_50_ value of THI was 155.4 mg/L (Figure S1). Subsequently, the turtles were randomly assigned to three groups for subsequent hepatotoxicity assessments: the high‐concentration group at 15 mg/L (THI‐H, approximately one‐tenth of the LC_50_); an environmentally relevant concentration group at 4.5 μg/L (THI‐EC; Süß et al. 2006); and a control group without THI. The turtles were maintained in water containing THI for a period of 5 weeks. Then turtles were euthanized using ethyl carbamate, and liver samples were extracted and stored at −80°C. Liver samples from five individuals per group were utilized for transcriptome sequencing analysis, oxidative stress enzyme activity assays, and quantitative real‐time polymerase chain reaction (qRT‐PCR) analysis. Additionally, liver samples from three individuals per group were fixed with paraformaldehyde for the TdT‐mediated dUTP Nick‐End labelling (TUNEL) assay.

### 2.3 RNA Sequencing Analysis

Total RNA was extracted from liver tissue samples using the Trizol method (Huang et al. 2021). Briefly, 100 mg of liver tissue was weighed and 1 mL of Trizol and 2 grated beads were added. After grinding with a freezing mill, chloroform was added to extract, and the supernatant was centrifuged. Isopropanol was added and precipitated at −20°C. The precipitate was washed by adding 75% ethanol, and after ethanol was volatilized, DEPC water was added to dissolve the RNA. The integrity, concentration, and purity of the RNA were evaluated through the use of agarose gel electrophoresis and Nanodrop 2000 (Thermo Fisher, USA), respectively. A total of 1 μg RNA was used for each library construction. The libraries were prepared using the Mumina NovaSeq Reagent Kit, and sequencing was conducted on the Illumina MiSeq platform. The statistical analysis of the raw sequence data includes: (1) A/T/G/C base content distribution statistics, (2) base quality distribution statistics, and (3) base error rate distribution statistics. Quality control is performed on the raw data to obtain high‐quality sequencing data (clean data): (1) remove adapter sequences from reads, and remove reads without insert fragments due to adapter self‐connection or other reasons; (2) trim the base with low quality (quality value < 20) at the end of the sequence (3′ end), delete the whole sequence if there is still a quality value < 10 in the remaining sequence, otherwise keep the sequence; (3) the reads with more than 10% N content were removed; and (4) the sequences < 20 bp in length after adapter and quality pruning were discarded. The collated quality control data were employed in the analysis of expression levels. Gene ontology (GO) enrichment analysis was performed using Goatools. Fisher's exact test was applied to identify significant enrichment among differentially expressed genes (DEGs) after adjusting for multiple comparisons. A *p*‐value of < 0.05 was considered to indicate significant enrichment. The sequences have been deposited in GenBank with the accession number PRJNA1072973.

### 2.4 Oxidative Stress Enzyme Activity Assay

Liver tissue samples were homogenized using a low‐temperature, high‐throughput tissue grinder (Ningbo Xinzhi Biotechnology Co. Ltd., Ningbo, China) on 100 mg aliquots. The resulting homogenate was then subjected to centrifugation at 4°C for 10 min at 1780 *g*, after which the supernatant was transferred to a new EP tube. The BCA protein assay kit from Beyotime Biological Company (Shanghai, China) was utilized to determine the concentrations of liver proteins. The activities of catalase (CAT), glutathione peroxidase (GPX), superoxide dismutase (SOD), and malondialdehyde (MDA) were quantified using kits from the Nanjing Jiancheng Institute of Biotechnology (Nanjing, China). The activity of CAT was evaluated through the observation of the direct decomposition of hydrogen peroxide (H_2_O_2_) under defined conditions, which resulted in a gradual decline in its concentration and a concomitant reduction in absorbance. MDA, a degradation product of lipid peroxidation, reacts with thiobarbituric acid (TBA) to form a red complex, the absorbance of which was measured at 532 nm. The superoxide anion radical, generated by the xanthine and xanthine oxidase reaction system, oxidizes hydroxylamine to form nitrite, yielding a purple‐red complex that can be measured at 550 nm. Furthermore, the reaction between GSH and dithio‐dinitrobenzoic acid (DTNB) results in the formation of a yellow anion of 5‐thio‐2‐nitrobenzoic acid, which exhibits stable colouration and was measured at 412 nm.

### 2.5 Caspases Activity Assay

Liver tissue samples, each with a mass of 100 mg, were pulverized using a cryogenic high‐throughput tissue grinder. The homogenized samples were subjected to centrifugation at 11,170 *g* for a period of 5 min at 4°C, after which the supernatant was carefully transferred to fresh EP tubes. The protein concentration in the liver tissue homogenates was determined using the Bradford assay (Servicebio, Wuhan, China). The activities of caspase‐3, caspase‐8, and caspase‐9 were quantified using assay kits provided by Jiangsu Kaiji Biotechnology Co. Ltd. (Jiangsu, China). Each well was supplied with 50 μL of reaction buffer and 5 μL of substrate, and was then incubated for 4 h at 37°C in the dark. The optical density (OD) values were recorded at 405 nm using a microplate reader. The extent of caspase activation in the experimental groups was calculated as the ratio of OD values of the treated samples to those of the negative controls.

### 2.6 qRT‐PCR Analysis

Total RNA was extracted using the same methods as in Section 2.3. The integrity and purity of the RNA were evaluated through the use of agarose gel electrophoresis and Nanodrop (Thermo, USA), respectively. Reverse transcription was conducted using the FastKing cDNA First Strand Synthesis Kit from TIANGEN Biochemical Technology Co. Ltd. (Beijing, China). Primers for the target genes (Table S1) were designed based on the NCBI sequences and synthesized by Sangon Biotech Co. Ltd. (Shanghai, China). The specific gene include glucose regulated protein (GRP78), activating transcription factor 6 (ATF6), activating transcription factor (ATF4), inositol‐requiring enzyme 1α(IRE1α), eukaryotic translation initiation factor 2α (eIF2α), X‐box binding protein 1 (XBP1), C/EBP homologous protein gene (CHOP), c‐Jun N‐terminal kinase (JNK), protein kinase R‐like endoplasmic reticulum kinase (PERK), b‐cell lymphoma‐2 (Bcl‐2), bcl‐2 associated X protein (Bax), cysteinyl aspartate specific proteinase (Caspase3), and β‐actin. Quantitative real‐time PCR (qRT‐PCR) was conducted using a LightCycler 480 instrument (Roche Diagnostics) with Genious 2 × SYBR Green Fast qPCR Mix (ABclonal, China) as the fluorescent dye. The experimental conditions were as follows: predenaturation at 95°C for 3 min; annealing and extension: 40 cycles at 95°C for 5 s; 60°C, 30 s. The relative expression levels of the genes were determined using the $2^{-\Delta \Delta C_{t}}$ method.

### 2.7 TUNEL Assay

The fixed liver samples were dehydrated through a graded series of alcohol, cleared with xylene, and embedded in paraffin. Sections of 5 μm thickness were prepared using a Leica CM1950 microtome (Hesse, Germany). Following the removal of xylene and rehydration through a graded alcohol series, the samples were treated with Proteinase K working solution and incubated at 37°C. Following membrane permeabilization, the samples were incubated with fluorescein isothiocyanate (FITC). The nuclei were counterstained with DAPI, and an anti‐fade mounting medium was applied. Fluorescence microscopy was utilized for the observation and documentation of the stained sections.

### 2.8 Data Analysis

The data are presented as mean ± standard error of the mean (SEM). Statistical comparisons among groups were performed using one‐way analysis of variance (ANOVA) followed by LSD's multiple comparison test, after confirming the data met assumptions of normality and homogeneity of variance. The statistical analyses were conducted using the SPSS software version 20.0, with a significance level of *p* < 0.05.

## 3 Results

### 3.1 Transcriptome Analysis of the Liver in THI‐Exposed Turtles

**FIGURE 1:**
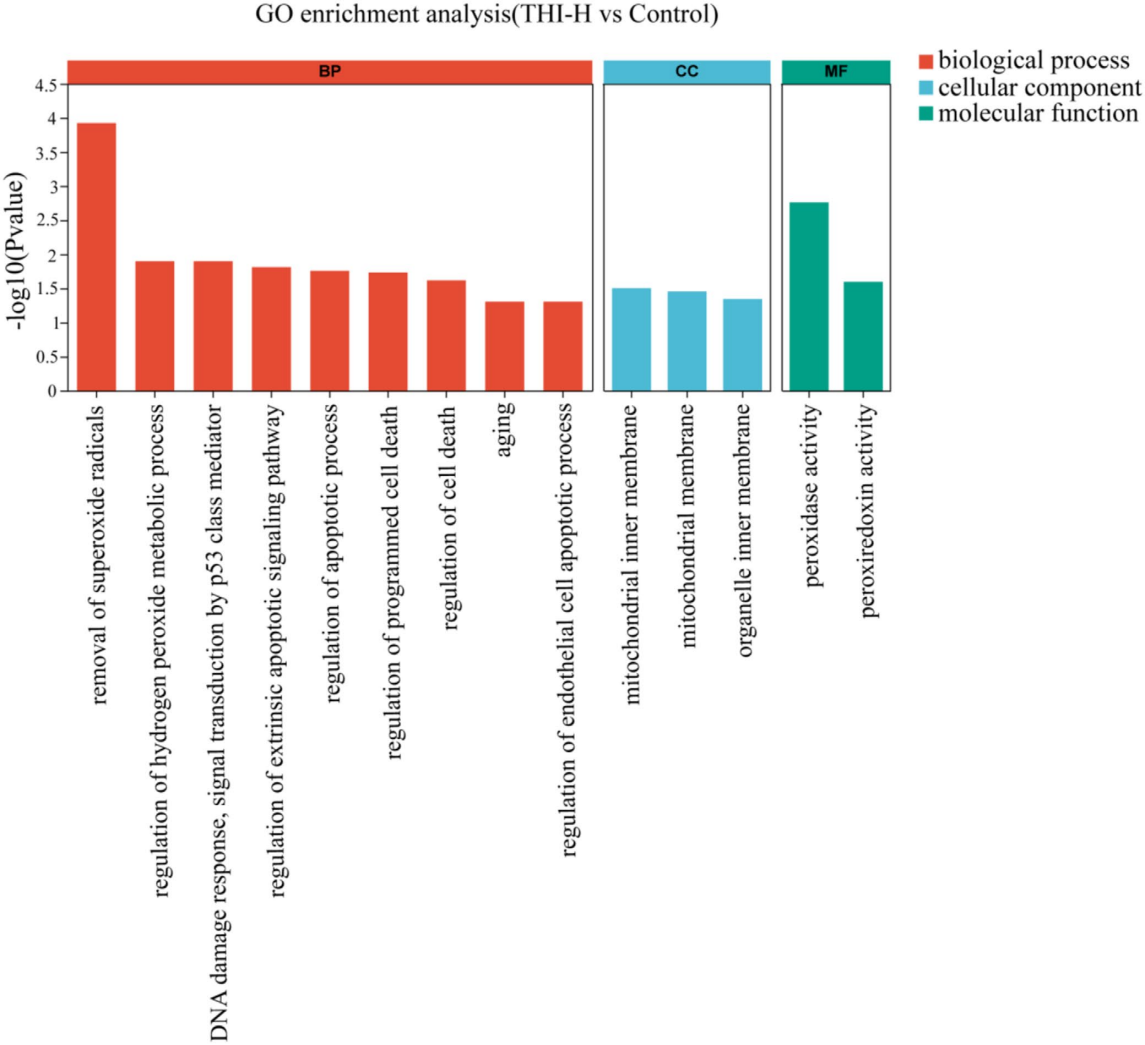
The effects of THI on the liver transcriptome in turtles. GO classification maps of the THI‐H and control groups.

The GO enrichment analysis revealed that the high‐concentration THI exposure group (THI‐H) was predominantly enriched in biological process (BP) pathways associated with the removal of superoxide radicals and apoptosis, as compared to the control group. The cell component (CC) was predominantly concentrated in the mitochondrial inner membrane and organelle inner membrane. Additionally, peroxiredoxin activity was primarily enriched in the molecular function (MF) category (Figure 1).

### 3.2 THI‐Induced Oxidative Stress in the Liver

**FIGURE 2:**
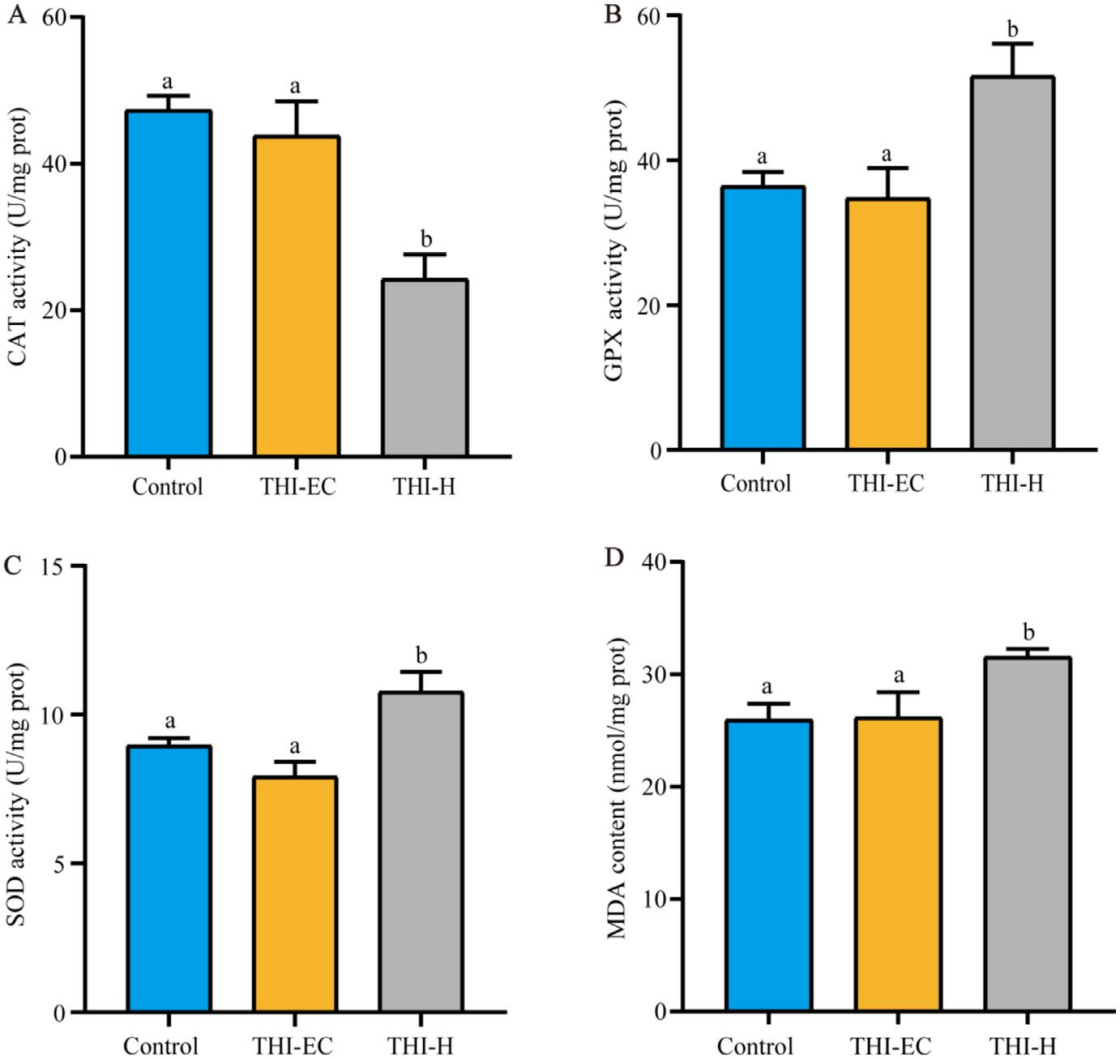
THI exposure induced oxidative stress in turtles. (A) CAT activity. (B) GPX activity. (C) SOD activity. (D) MDA content. Data are expressed as mean ± SEM. Different letters indicate statistically significant differences between different treatment groups (*p* < 0.05).

A significant reduction in CAT activity was observed in the high‐concentration group, with a 48.72% decrease (Figure 2A). Concurrently, there was a significant increase in GPX and SOD activities by 41.68% and 20.25%, respectively (Figure 2B,C). Furthermore, MDA levels exhibited a notable increase of 21.52% (Figure 2D).

### 3.3 THI‐Induced ER Stress in Liver

**FIGURE 3:**
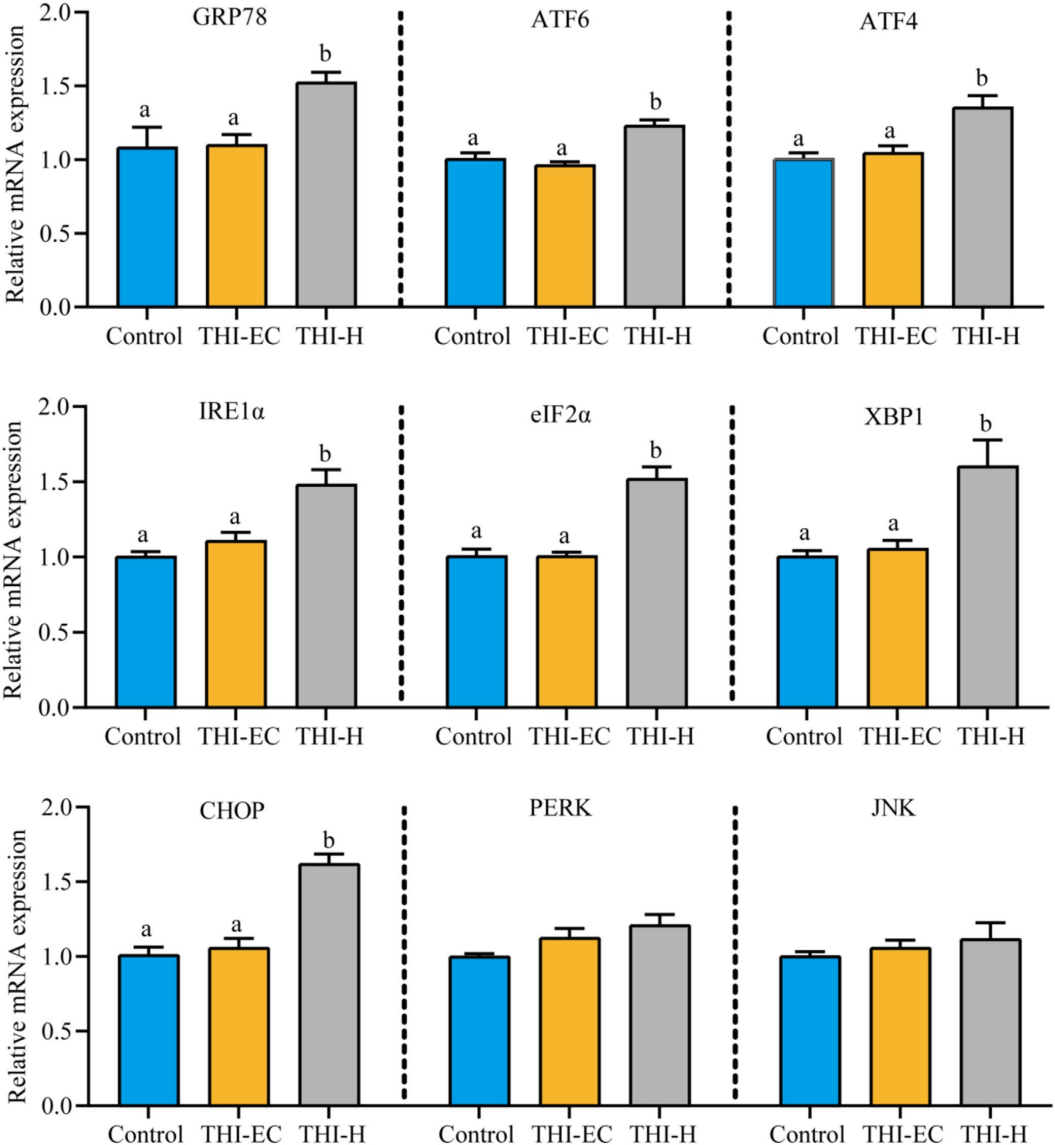
THI exposure caused ERS in turtles. Data are expressed as mean ± SEM. Values not sharing a common superscript letter differ significantly at *p* < 0.05.

In the group subjected to high concentrations of THI, the expression levels of GRP78, ATF6, ATF4, IRE1α, eIF2α, XBP1, and CHOP genes were significantly elevated compared to the control group. Additionally, the transcriptional levels of JNK and PERK were observed to increase following exposure to high concentrations of THI, although this increase did not reach statistical significance (Figure 3).

### 3.4 THI Exposure Induced Apoptosis in the Liver of Turtles

**FIGURE 4:**
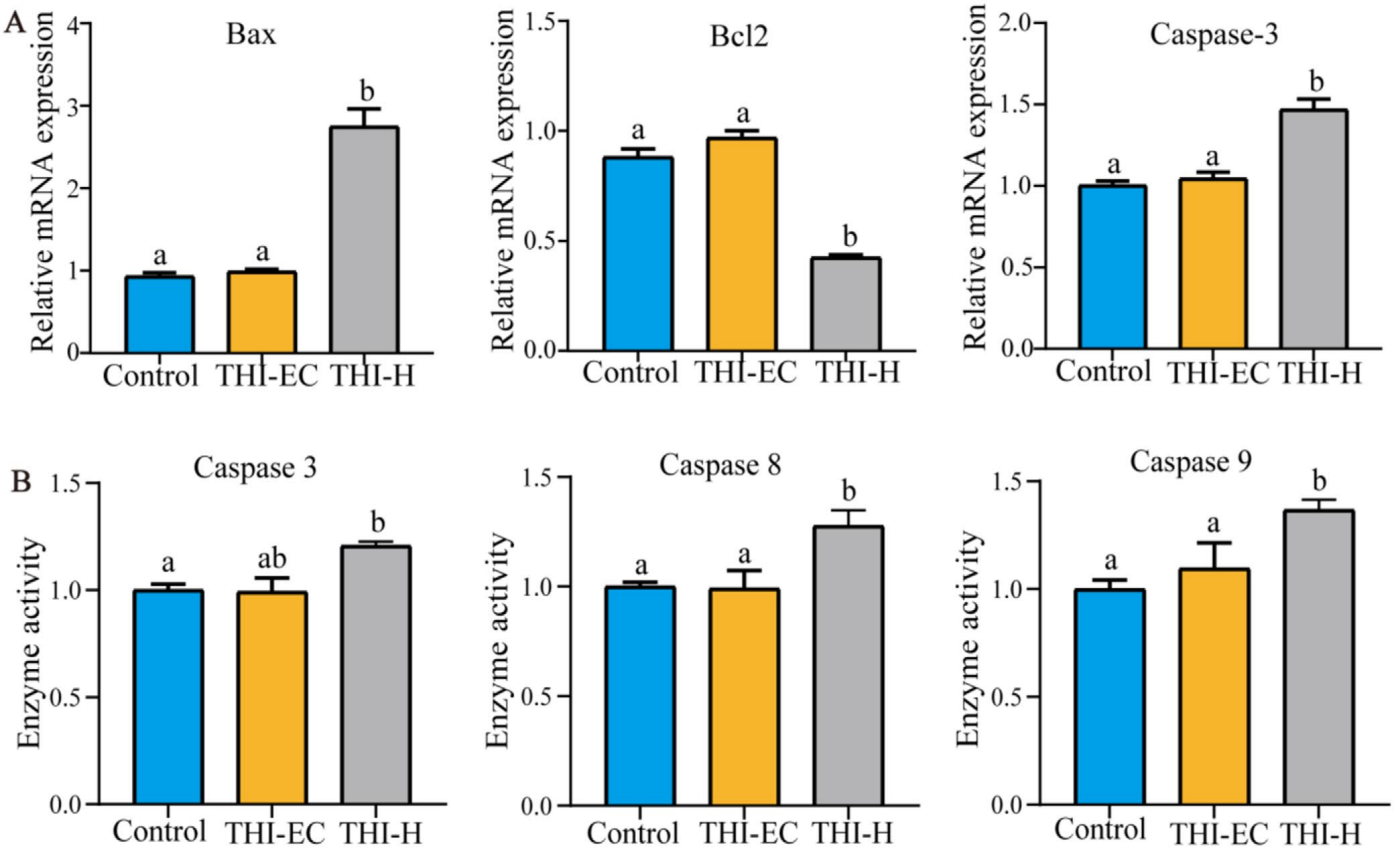
THI exposure promoted apoptotic pathways in the liver. (A) Relative mRNA expression of Bax, Bcl‐2, caspase‐3. (B) Activities of caspase‐3, caspase‐8, and caspase‐9 of turtle liver. Data are expressed as mean ± SEM. Values not sharing a common superscript letter differ significantly at *p* < 0.05.

**FIGURE 5:**
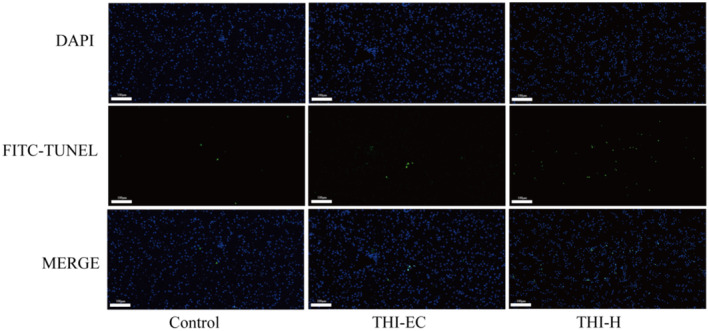
Cell apoptosis in the liver in THI exposed turtles. The first row shows nuclei (blue), the second row shows apoptosis‐positive cells (green), and the third column shows merging.

Compared to the control group, the mRNA expression levels of Bax and caspase‐3 were found to be significantly elevated, while the expression of Bcl‐2 in the liver of the THI‐H exposure group was observed to be significantly reduced (Figure 4A). Moreover, the activation levels of caspase‐3, caspase‐8, and caspase‐9 in the THI‐H group were found to be significantly elevated in comparison to the control group (Figure 4B). In addition, hepatocyte apoptosis was assessed using the TUNEL method. The results indicated that while the number of positive cells in the THI‐EC group did not increase significantly, there was a marked increase in the number of positive cells in the THI‐H group (Figure 5).

## 4 Discussion

Wild turtles often live in lakes, reservoirs, ponds, swamps, rice paddies, and other waters, and most of them are omnivorous. In nature, they feed mainly on worms, small fish, shrimps, snails, earthworms and animal carcasses, and may also eat duckweed, water grass, rice, plant stems, leaves, melons, and other plants. Due to the widespread use of THI in agriculture and characteristics of accumulation and biomagnification, turtles can then easily be exposed to neonicotinoids in a variety of ways, such as direct contact with bodies of water and indirect contact through food chains. However, there are no reports on the effects of neonicotinoid insecticides on turtles. THI has a long history of use and is highly water soluble, which inevitably causes harm to nontarget organisms in the environment, especially aquatic organisms (Yan et al. 2015; Morrissey et al. 2015). The LC_50_ of THI was 80.24 mg/mL in zebrafish (Xie et al. 2022) and 640 mg/kg in rats (Sheets et al. 2016). In terms of acute toxicity to crustaceans, imidacloprid and THI are the most toxic (Morrissey et al. 2015). Finnegan et al. (2017) demonstrated the acute and chronic toxicity of thiamethoxam to aquatic organisms, including aquatic plants, aquatic invertebrates, and fish, with the median acute lethal concentration (LC_50_) ≥ 80 mg/L in all cases. The study unequivocally demonstrates that the 96 h LC_50_ of THI against turtles was 155.4 mg/L. This suggests that there are differences in the susceptibility of different animal species to neonicotinoid insecticides, with turtles being more tolerant to THI than other aquatic animals and more susceptible to THI than mammals.

The liver is the major detoxification organ in animals and serves as a sensitive barometer for the extent of damage inflicted by environmental pollutants. To elucidate the effects of THI on turtles and to uncover the underlying mechanisms, we initiated our investigation with a comprehensive transcriptome analysis. In this study, GO enrichment analysis predominantly highlighted pathways associated with superoxide radical scavenging, peroxiredoxin activity, organelle inner membrane integrity, and apoptosis. Previous research has demonstrated that neonicotinoids can disrupt physiological and biochemical systems, as well as antioxidant defenses (Yan et al. 2016). THI has been shown to induce lipid peroxidation, dysregulation of antioxidant enzyme levels, and ERS in fish, culminating in hepatocyte toxicity (Velisek and Stara 2018; Xie et al. 2022). These observations are consistent with our transcriptomic data, further substantiating the biological perturbations induced by THI.

Oxidative stress plays a central role in the aging process and in the etiology of several diseases, and its deleterious effects on biological systems are well documented. H_2_O_2_, capable of permeating most cellular membranes, poses a cytotoxic threat. The CAT enzyme mitigates the toxicity of H_2_O_2_ by decomposing it into oxygen and water, thereby serving as a cornerstone of biological antioxidant defense mechanisms (Ge et al. 2015). SOD, a metalloenzyme, combats oxidative stress by neutralizing free radicals. An excess of free radicals typically triggers an increase in SOD activity. Oxygen radicals can attack the polyunsaturated fatty acids (PUFAs) in the cell membrane, initiating lipid peroxidation and the formation of lipid peroxides such as MDA, which are detrimental to cellular integrity (Martins, Leão, and Vasconcelos 2009). Consequently, MDA serves as a biomarker of oxidative damage to cell membranes (Martins, Leão, and Vasconcelos 2009). GPX specifically catalyzes the reduction of hydrogen peroxide by reduced glutathione (GSH), thereby protecting the structure and function of the cell membrane. Exposure to imidacloprid has been shown to induce oxidative stress in the liver and brain of rats (Duzguner and Erdogan 2012) and to disrupt the antioxidant defense system of earthworms ( *Eisenia fetida* ), leading to lipid peroxidation (Zhang, Zhang, and Wang 2014). Furthermore, in the tropical fish *Prochilodus lineatus* , imidacloprid exposure has been associated with changes in antioxidant enzyme activity, culminating in hypoglycaemia and DNA damage (Vieira et al. 2017). In our study, we quantified the activities of CAT, SOD, and GPX, as well as MDA levels, to evaluate the oxidative stress in the liver. The THI‐H group had significantly reduced CAT levels and increased MDA, SOD, and GPX levels compared to the control group. These results suggest that although turtles possess intrinsic defenses against toxin‐induced oxidative stress, the observed decrease in CAT and the concomitant increase in MDA levels indicate a compromised antioxidant defenses and the presence of oxidative stress.

The endoplasmic reticulum (ER) is the membrane system of the cytoplasm that is mainly responsible for the synthesis of proteins and lipids and the transport of intracellular substances (Oyadomari and Mori 2004). ER stress is a protective response activated by cells in the face of misfolded and unfolded protein aggregation and calcium ion imbalance in the endoplasmic reticulum lumen. GRP78, also known as Bip, is a key cellular companion protein that helps regulate the folding of other cellular proteins and is ERs marker molecule (Liu et al. 2023). Previous studies have shown that GRP78 binds to parts of the ER chambers of PERK, IRE1, and ATF6 receptors in the unstressed state, when the protein receptors are inactive. When the protein aggregates in the ER cavity and the ER is in a stressed state. GRP78, which has a strong binding ability to unfold proteins, dissociates and is released into the ER cavity to perform protein folding functions, activating the ER receptor. Three major signaling pathways XBP1, eIF2a, and ATF6, generate ERs response elements (ERSE) and promote the unfolded protein response (UPR) by inducing transcription of multiple chaperones at the transcriptional level. This can enhance the ability of the ER to remove unfolded or misfolded proteins and reduce protein synthesis to regulate the environmental balance in the ER (Martins, Leão, and Vasconcelos 2009). Prolonged or excessive ER can cause irreversible damage to cells (Logue et al. 2013). A study in gibel carp (*Carassius auratus gibelio*) showed that gibberellic acid caused liver damage by inducing oxidative stress, ERs, and apoptosis (Ma 2023). In our study, transcript levels of GRP78, ATF6, ATF4, and eIF2α were significantly upregulated during THI exposure, suggesting that ER balance was disrupted and the involvement of an UPR restored ER balance to protect the body from further damage during THI exposure. If ER dysfunction persists, cells will then eventually initiate an apoptotic programme (Logue et al. 2013).

Increasing evidence has implicated oxidative stress and ER stress as key contributors to the induction of apoptosis (Jia et al. 2019; Hu et al. 2022). Under conditions of excessive ER stress, the pro‐apoptotic branch of the UPR is activated, which in turn triggers apoptotic signaling in the cell. IRE1, an ER‐resident protein kinase, plays a key role in this process. As the accumulation of unfolded proteins in the ER escalates, the IRE1‐BIP/GRP78 complex dissociates, leading to the activation of IRE1 following oligomerization and autophosphorylation (Huang et al. 2021). Activated IRE1 modulates cell survival and apoptosis signaling. During apoptosis, IRE1‐activated c‐Jun N‐terminal kinase (JNK) phosphorylates and inhibits the activity of anti‐apoptotic Bcl‐2 family proteins, thereby promoting cell death. Concurrently, caspase‐12 is activated, initiating the caspase cascade and mediating apoptosis (Yoneda et al. 2001). Furthermore, IRE1 has ribonuclease activity that cleaves XBP1 mRNA, facilitating its maturation and enhancing the transcriptional expression of molecular chaperone proteins and CHOP, thus facilitating apoptosis (Yoneda et al. 2001). The ER stress response can also activate the PERK and ATF6 pathways, which further induce apoptosis (Jia et al. 2019; Huang et al. 2021; Hu et al. 2022; Cao et al. 2022). In the context of THI‐exposed turtles, we observed upregulation in the transcriptional levels of IRE1α, XBP1, and CHOP, leading us to infer that persistent disruption of endoplasmic reticulum function may enhance apoptotic signaling.

Apoptosis is a type of programmed cell death that is triggered by signals generated within the cell. Various stresses, including DNA damage, oxidative stress, and loss of survival signals, cause mitochondrial outer membrane permeabilization (MOMP) (Eriten et al. 2024). MOMP causes the release of cytochrome c into the cytosol, where it binds to apoptotic protease‐activating factor‐1 (Apaf‐1) to form the apoptosome. The apoptosome then activates caspase‐9, which in turn activates effector caspases such caspase‐3, resulting in the deconstruction of cellular components and cell death (Emre Kızıl et al. 2023). Members of the Bcl‐2 protein family strictly regulate the intrinsic pathway, which includes pro‐apoptotic proteins such as Bax and Bak as well as anti‐apoptotic proteins like as Bcl‐2 and Bcl‐xL, to maintain a balance between survival and apoptosis (Varışlı et al. 2023). Previous studies reported that exposure to imidacloprid caused apoptosis of hepatocytes in grass carp ( *Ctenopharyngodon idella* ) via the intrinsic apoptosis pathway (Miao et al. 2022). We found that Bax and caspase‐3 gene expression levels were increased, Bcl‐2 expression was decreased, and caspase‐9 and caspase‐3 enzyme activities were upregulated, indicating that the endogenous apoptotic pathway was promoted. It is worth noting that in addition to the intracellular apoptotic pathway, apoptosis can also occur via the extracellular pathway, namely the death receptor pathway (Gupta 2001). Activation of caspase‐8 and downstream caspase‐3 are typical extracellular apoptotic pathways (Yamashita 2003). In the present study, the extrinsic apoptotic pathways caspase‐8 and caspase‐3 were activated and upregulated.

## 5 Conclusion

Our research findings indicate that exposure to THI leads to a significant enrichment of pathways associated with the clearance of superoxide radicals, peroxiredoxin activity, the integrity of organelle inner membranes, and apoptosis in turtles, as confirmed through transcriptome analysis. This exposure triggers oxidative stress, which is manifested by changes in the activity of antioxidant enzymes, and subsequently activates the UPR within the turtle liver. Moreover, both exogenous and endogenous apoptotic pathways are upregulated in response to THI exposure, ultimately inducing apoptosis in the affected turtles.

## Author Contributions

**Shuqin Lin:** conceptualization (equal), data curation (equal), formal analysis (equal), investigation (equal), methodology (equal), software (equal), validation (equal), visualization (equal), writing – original draft (equal). **Yunjuan Xiao:** methodology (equal), software (equal), validation (equal). **Siyu Li:** methodology (equal). **Liyan Tang:** methodology (equal). **Haitao Shi:** supervision (equal). **Meiling Hong:** writing – review and editing (equal). **Li Ding:** conceptualization (equal), funding acquisition (equal), investigation (equal), project administration (equal), resources (equal), supervision (equal), validation (equal), writing – review and editing (equal).

## Conflicts of Interest

The authors declare no conflicts of interest.

## Supporting information


Figure S1.

Table S1.

## Data Availability

Liver transcriptome sequences are available at NCBI (login Quantity: PRJNA853921).
