# Supplementary figures and images for: Thiacloprid Exposure Induces Oxidative Stress, Endoplasmic Reticulum Stress, and Apoptosis in the Liver of Mauremys reevesii
Source: Ecol Evol. 2025 Feb 4;15(2):e70936. doi: 10.1002/ece3.70936 (PMC11794825; doi:10.1002/ece3.70936)

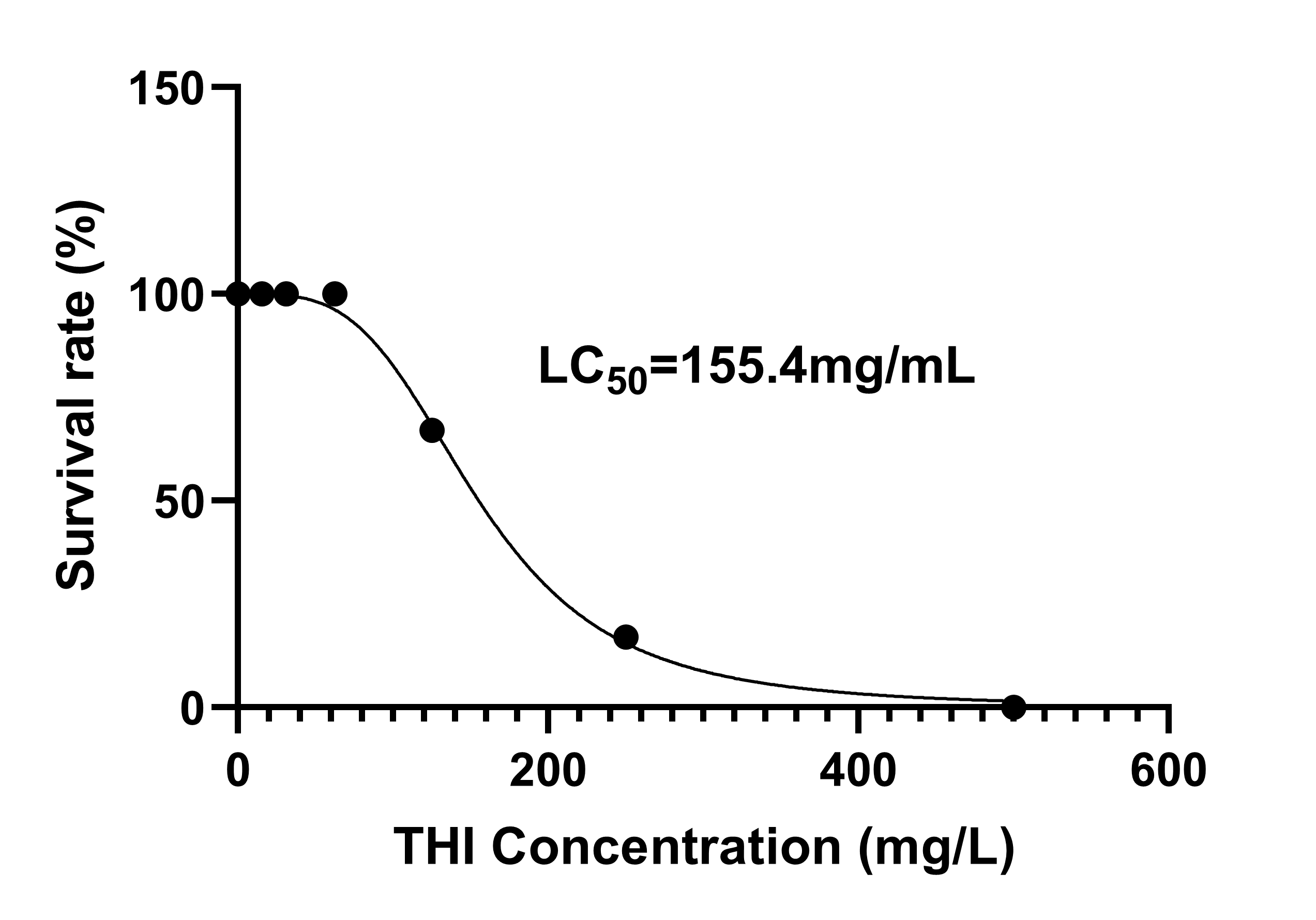

Supplement: Supplementary file 1 — Figure S1. [file ECE3-15-e70936-s002.tif]
