# Supplementary material for: Thiacloprid Exposure Induces Oxidative Stress, Endoplasmic Reticulum Stress, and Apoptosis in the Liver of Mauremys reevesii
Source: Ecol Evol. 2025 Feb 4;15(2):e70936. doi: 10.1002/ece3.70936 (PMC11794825; doi:10.1002/ece3.70936)
Supplement: Supplementary file 2 — Table S1. [file ECE3-15-e70936-s001.docx]

**Table**

Table S1. The primer sequences used in the this study.

| Gene | Primer sequence (5'-3') |
| --- | --- |
| GRP78 | F: CCTGGTGCTACTTGACGTGT  R: TCAATCTGTGGGACACCACG |
| ATF6 | F: GTTTCTCCACCACCCTGTCA  R: CTGATGCTGGGGTCTGTGTT |
| ATF4 | F: AGTGGTCTTTGGTGGCTGAG  R: AGAACCCATGCGAACAGAGG |
| IRE1α | F: TGCAGCCACTGTACCTGATG  R: AAAAGCTACCACTGGGGACC |
| eIF2α | F: TTCCAAGCTGCATGAGGAGG  R: GTGTTGATTCCTGGCTCCCA |
| XBPX1 | F: CCTGGACCCAGACATGTTCC  R: CTTTTCTGCAGATTTCTTCCTCA |
| CHOP | F: CAGAGCTCCCCAGCTCAGAC  R: TTCTCTTCCTCTTCACGCCG |
| PERK | F: CCCCAAATACCCCCAGTCAT  R: TTCTTTCTCTGGCACACAGGG |
| JNK | F: CACTGGTTACTGCACGCCTA  R: GCAGGTACCAAAGCCAACAG |
| BAX | F: CGGTCTCGAAGGAGGTGTTT  R: TCCTGAGCGATGACCCTGTA |
| BCL-2 | F: TGCTAGTAACGTGCCCCTTG  R: TCATCTCCAGCTTGGCACAG |
| Caspase-3 | F: TGGACTGCAATCAGGTCACG  R: ACAGGCAGGCTCTTTCCTTG |
| β-actin | F: CCGTGATCTGACGGACTACC  R: TGTCACGCACGATTTCCCTT |
